# A fusion protein of vimentin with Fc fragment inhibits Japanese encephalitis virus replication

**DOI:** 10.3389/fvets.2024.1368725

**Published:** 2024-03-04

**Authors:** Taoping Zhang, Zhixin Chen, Lyu Xie, Ruixian Xu, Lu Chen, Ting Jia, Wengang Shi, Yongbo Wang, Yuzhu Song, Qinqin Han, Xueshan Xia, Tao Yuan, Jinyang Zhang

**Affiliations:** ^1^Faculty of Life Science and Technology, Kunming University of Science and Technology, Kunming, China; ^2^Yunnan Provincial Key Laboratory of Clinical Virology, The First People’s Hospital of Yunnan Province, Kunming, China; ^3^Yunnan Province Clinical Research Center for Gynecological and Obstetric Disease, Kunming, China; ^4^Institute of Basic Medical Sciences, School of Basic Medicine, Peking Union Medical College, Chinese Academy of Medical Sciences, Beijing, China; ^5^Department of Clinical Laboratory, The First People’s Hospital of Yunnan Province, Kunming, China; ^6^Department of Gynecology, The First People’s Hospital of Yunnan Province, Kunming, China

**Keywords:** JEV, vimentin, Fc fragment, fusion protein, antiviral

## Abstract

Japanese encephalitis virus (JEV), a member of the Flaviviridae family and a flavivirus, is known to induce acute encephalitis. Vimentin protein has been identified as a potential receptor for JEV, engaging in interactions with the viral membrane protein. The Fc fragment, an integral constituent of immunoglobulins, plays a crucial role in antigen recognition by dendritic cells (DCs) or phagocytes, leading to subsequent antigen presentation, cytotoxicity, or phagocytosis. In this study, we fused the receptor of JEV vimentin with the Fc fragment of IgG and expressed the resulting vimentin-Fc fusion protein in *Escherichia coli*. Pull-down experiments demonstrated the binding ability of the vimentin-Fc fusion protein to JEV virion *in vitro*. Additionally, we conducted inhibition assays at the cellular level, revealing the ability of vimentin-Fc protein suppressing JEV replication, it may be a promising passive immunotherapy agent for JEV. These findings pave the way for potential therapeutic strategies against JEV.

## 1 Introduction

Japanese encephalitis virus (JEV) is a member of the Flaviviridae family, specifically classified as a flavivirus. The RNA of JEV consists of a single strand with a length of approximately 11 kb (1). The discovery of JEV occurred in Japan during the 1970s, and it is recognized as an acute and severe infectious disease of the central nervous system. Humans and animals transmit the virus to one another through mosquito vectors (2, 3). The clinical manifestations of this disease primarily include high fever, transient generalized or localized limb convulsions, sensory impairments, and meningeal damage (4–6). In 1924, JEV was first isolated in the laboratory, revealing a spherical shape with an average virus diameter of about 40 nanometers. The virus is composed of viral envelope proteins and nucleic acids, where the outer layer consists of an envelope containing viral hemagglutinins and protrusions of viral glycoprotein (E) (7). The viral inner membrane protein (M), involved in virus assembly, is located inside the envelope. Although substantial progress has been made in understanding the pathogenesis of JEV, including the structure and function of protein nucleic acids, the receptor protein for JEV remains incompletely studied, leaving certain aspects of its pathogenicity still enigmatic. The main infectious source of domestic animals infected with JEV is pigs, which can be infected by both adult and young pigs (8), and the application of vaccine has not reached the expectation, and there are still many defects of fire-extinguishing and attenuated vaccines (9, 10), which bring difficulties in the prevention and treatment of JEV. Therefore, the development of JEV antivirals and research into the pathogenesis of JEV are still the key points.

Vimentin, an important intermediate filament protein in the cellular cytoskeleton (11), serves as a crucial receptor for JEV. It is a cell surface molecule that exhibits extensive interaction with JEV viral particles (12). As one of the most important intermediate filament proteins, vimentin is expressed in certain ectodermal and mesenchymal cells. To maintain cellular integrity, vimentin forms a network of cytoskeletal scaffolding along with microtubules and microfilaments. Vimentin participates in regulating mechanisms of cell–cell interactions, resulting in different phosphorylation patterns across various cellular states (13, 14). In addition to preserving cell integrity, vimentin also influences cell adhesion, migration, apoptosis, and inflammation (15–18). Moreover, vimentin plays an intermediary role in multiple viral infection processes and has been found to be positively correlated with human enterovirus EV7 (19).

The Fc fragment, a crucial region of immunoglobulins, plays a pivotal role in antigen processing upon complex formation with antibodies. Composed of 226 amino acids, it exerts a significant impact on various biological functions of antibodies and participates in immunoglobulin-mediated pathological damage or physiological processes. The Fc fragment can be recognized by dendritic cells (DCs) or phagocytes, facilitating the killing or phagocytosis of the antigen-bearing cells (20, 21). Fusion proteins containing the Fc domain possess both the properties of immunoglobulins and the properties of the protein of interest. Among the proteins found in human blood, IgG is the most abundant, boasting an extended half-life of up to 21 days (22). The Fc fragment, classified as a soluble protein, can also enhance the solubility of other proteins when expressed as fusion constructs.

In this research, a new perspective is presented for the development of therapeutics against JEV. The study involves the fusion of the potential receptor vimentin with the Fc fragment, resulting in the expression and purification of the vimentin-Fc fusion protein. By conducting pull-down experiments, the interaction between the fusion protein and the virus was identified. Moreover, cellular experiments demonstrated the inhibitory effects of the vimentin-Fc fusion protein on JEV replication. These novel findings pave the way for innovative strategies in the development of antiviral drugs against JEV.

## 2 Materials and methods

### 2.1 Materials

Vero, BHK-21 and C6/36 were originally purchased from the Chinese Typical Culture Preservation Center and the Kunming Cell Bank of the Chinese Academy of Sciences, corresponding to the numbers GDC0029, GDC0010, and KCB82002YJ, and have been stably stored in our laboratory for many years. SP2/0 cells were preserved by this laboratory.

JEV was isolated from the live attenuated vaccine strain JEV-SA14-14-2 of Wuhan Keqian Animal Biology Co. Hybridoma cell line 3C4 was screened and prepared in this study. BALB/c mice were purchased from the Animal Experiment Centre of Kunming Medical University. The animal experiments was approved by the Animal Care and Use Committee of Kunming University of Science and Technology.

### 2.2 Preparation of monoclonal antibodies against JEV

The preparation of monoclonal antibodies involved the collection of supernatant from JEV culture supernatants after 3 days of cultivation. The supernatant was then centrifuged at 12,000 rpm/min to remove impurities. Three-days-old BALB/c mice were selected for immunization by intracranial injection of the cultured JEV. After 7 days, the mouse brains were dissected in a biosafety cabinet and ground using a mortar and pestle. The ground brain tissue was suspended in DMEM basal culture medium and subsequently centrifuged at 4°C, 2,500 × g for 15 min using a refrigerated centrifuge. The resulting supernatant was divided into Eppendorf tubes, followed by 15 min of UV irradiation for inactivation. This JEV brain supernatant served as the antigen for immunizing the mice (23).

The prepared JEV brain supernatant was thoroughly mixed with Freund’s adjuvant. Subsequently, 300 μL of the mixture was injected subcutaneously at multiple sites on the backs of 6 weeks-old BALB/c mice. A total of three mice were immunized, with an immunization interval of 14 days and a total of five immunizations.

One mouse demonstrating a favorable immune response was selected for blood collection. The mouse was then euthanized, and the spleen was dissected within a biosafety cabinet. A grinder containing 5 mL of incomplete 1640 medium was used to grind the spleen, and subsequent rinsing of the grinder with 5 mL of incomplete 1640 medium was performed. The resulting cell suspension was transferred to a sterile 50 mL centrifuge tube. This process was repeated 2–3 times, and the cell suspension was supplemented with incomplete 1640 medium to a final volume of 40 mL. The SP2/0 cells in the logarithmic growth phase were collected in a 50 mL centrifuge tube and supplemented with incomplete 1640 medium to a final volume of 40 mL. After washing both types of cells three times with incomplete 1640 medium, cell fusion was performed. After approximately 2 weeks, the formation of cell clusters and the presence of positive hybridoma cells were observed using indirect immunofluorescence. Positive monoclonal clones were subcloned using the limiting dilution method, and the screening process was repeated three times to obtain positive hybridoma cells and subclones.

### 2.3 Specific validation of monoclonal antibodies against JEV

Vero cells were seeded in a 96 well plate at a density of 8 × 10^3^ cells per well and cultured overnight. When the cells reached 70%–80% confluency, they were infected with JEV at an MOI of 0.1 and incubated for 72 h. The cell morphology was observed during the incubation period, and uninfected cells were used as a negative control. Following fixation, the cells were blocked with 5% skim milk at 4°C overnight. Subsequently, an indirect immunofluorescence assay was performed using the selected monoclonal antibody (Anti-JEV-3C4) against JEV as the primary antibody and FITC-labeled goat anti-mouse IgG as the fluorescent secondary antibody. The unreacted solution was removed by washing with PBST, and the wells were kept moist. The fluorescence signals were visualized using an inverted fluorescence microscope.

BHK-21 cells were plated in a 6-well plate at a density of 1 × 10^6^ cells per well and incubated overnight. After 4 h of JEV virus infection at an MOI of 0.1, the medium was replaced with fresh medium containing 0.5% serum. After 72 h of cell culture, the cells were collected, and 200 μL of cell lysis buffer was added to each well for protein extraction. Uninfected cells were used as a negative control. The specificity of the monoclonal antibody was determined through Western blot analysis. The screened monoclonal antibody (Anti-JEV-3C4) specific to JEV was utilized as the primary antibody, while HRP-conjugated goat anti-mouse IgG was used as the secondary antibody. Visualization of the results was accomplished using the Easysee Western Blot Kit.

### 2.4 Construction of vimentin-Fc fusion vector

Lymphocytes were isolated from blood using cell separation medium and centrifugation. RNA extraction from the lymphocytes was then carried out using Trizol, followed by reverse transcription to obtain cDNA. Amplification of the vimentin and Fc genes was performed using specific primer sets (vimentin1-F, vimentin1-R, Fc1-F, and Fc1-R) (see Table 1). Subsequently, linker sequences were added to the vimentin and Fc genes using primers vimentin2-F, vimentin2-R, Fc2-F, and Fc2-R. The resulting vimentin-Fc gene was amplified using primers vimentin2-F and Fc2-R. To construct the pET-22b-vimentin-Fc recombinant expression vector, the vimentin-Fc gene was ligated with the pET-22b vector through double enzyme digestion.

**Table 1:** Primers list.

Vimentin1-F	ATGTCTACCAGGTCTGTGTCCTCGT
Vimentin1-R	TTCAAGGTCATCGTGATGCTGAG
Vimentin2-F	GGAATTCCAATGTCTACCAGGTCTGTGTC (*EcoR* I)
Vimentin2-R	CCCACCCCCGCCTGATCCTTCAAGGTCATCGTGATGCT
Fc1-F	GGTTGTAAGCCTTGCATATGTACAG
Fc1-R	TCATTTACCAGGAGAGTGGGAGA
Fc2-F	GGATCAGGCGGGGGTGGGGGTTGTAAGCCTTGCATATG
Fc2-R	ATAGTTTAGCGGCCGCTCATTTACCAGGAGAGTGGG (*Not* I)

### 2.5 Purification of the vimentin-Fc protein

The pET-22b-vimentin-Fc plasmid was transformed into *Escherichia coli* Rosetta, and the transformed cells were cultured in LB liquid medium. After that, IPTG was added to a final concentration of 0.1 mM to induce the expression of vimentin-Fc protein at 37°C for 4 h. The bacterial cells were harvested by centrifugation and resuspended in PBS. Cell disruption was achieved by ultrasound treatment, followed by centrifugation for 10 min. The resulting precipitate was denatured and dissolved in 8 M urea. The purified vimentin-Fc protein was then obtained using Ni-NTA affinity chromatography, following the manufacturer’s instructions. Finally, the protein preservation solution was exchanged with PBS solution through dialysis, and the protein was analyzed by SDS-PAGE and western blotting for identification.

### 2.6 Pull-down experiments

C6/36 cells were cultured in DMEM medium supplemented with 2% fetal bovine serum and then expanded with JEV-SA14-14-2 for 5 days. The culture supernatant from the inoculated cells and the supernatants from normal cells were collected as controls. Ni-NTA and protein A/G agarose beads were incubated and coupled with vimentin-Fc protein overnight at 4°C. Subsequently, the Ni-NTA and protein A/G agarose beads were washed with PBS. After washing, the culture supernatant from the inoculated cells and the normal cells were added and incubated at 25°C for 3 h. The Ni-NTA and protein A/G agarose beads were then washed with PBS again. RNA extraction from the Ni-NTA and protein A/G agarose beads was performed using the Trizol method, and cDNA was obtained through reverse transcription. PCR identification was conducted using JEV (M-E) primers (24):

Forward primer, 5′-TGATGACCATCAACAACACG-3′;

Reverse primer, 5′-CATGCGGACGTCCAATGTTG-3′.

The samples were analyzed by agarose gel electrophoresis for further characterization.

### 2.7 Cytotoxicity assay

BHK-21 cells were seeded in a 96-well plate at a density of 5 × 10^3^ cells per well. After 24 h of incubation, the cells were treated with vimentin-Fc fusion protein at concentrations ranging from 0.2 μg/mL to 100 μg/mL for 48 h at 37°C. Cells treated with PBS alone were used as the control. The cell toxicity of V + FC was evaluated using the CCK8 assay, incubating with CCK8 for 2 h and measuring the OD450. The experimental wells contained cells, culture medium, CCK8 solution, and fusion protein, while the control wells contained cells, culture medium, and CCK8 solution. The blank wells consisted of culture medium, CCK8 solution, without cells, and fusion protein. The calculation formula was [(experimental wells − blank wells)/(control wells − blank wells)] × 100%.

### 2.8 Inhibition experiment of vimentin-Fc protein in JEV-infected cells

To investigate the impact of vimentin-Fc protein on the interaction between the JEV and host cells during infection, we incubated BHK cells with wave vimentin-Fc protein or PBS before infection. After 4 h, JEV was added at a dosage of 0.01 MOI, and the cells were incubated for 3 h. Subsequently, the culture medium was replaced with fresh medium, and an equivalent amount of vimentin-Fc protein was added to the experimental wells. Cell culture supernatants were collected at 30 h, 54 h, and 78 h post-infection. RNA was extracted using a viral RNA extraction kit, followed by reverse transcription. The mRNA levels of JEV genes were quantified using SYBR real-time PCR. The PCR experiments were conducted on an ABI 7500 sequence detection system, and the expression levels of JEV genes were determined using the respective standard curve. Cell lysates were also collected for Western blot analysis to assess changes in viral protein.

### 2.9 Statistical analysis and data presentation

Unless stated otherwise, the raw data were graphed and statistically analyzed using GraphPad Prism 8 software (GraphPad Software). The data are presented as the mean ± standard error of the mean (SEM). For the given dataset, one-way analysis of variance (ANOVA) with multiple *t*-tests was conducted, and multiple comparisons were adjusted using the Holm-Sidak method to assess the significance of intergroup differences. Statistical significance was denoted by asterisks as follows: ^*^p < 0.05, ^**^p < 0.01, ^***^p < 0.001, and ^****^p < 0.0001.

## 3 Results

### 3.1 Predicted anti-JEV mechanism of vimentin-Fc fusion protein

**Figure 1:**
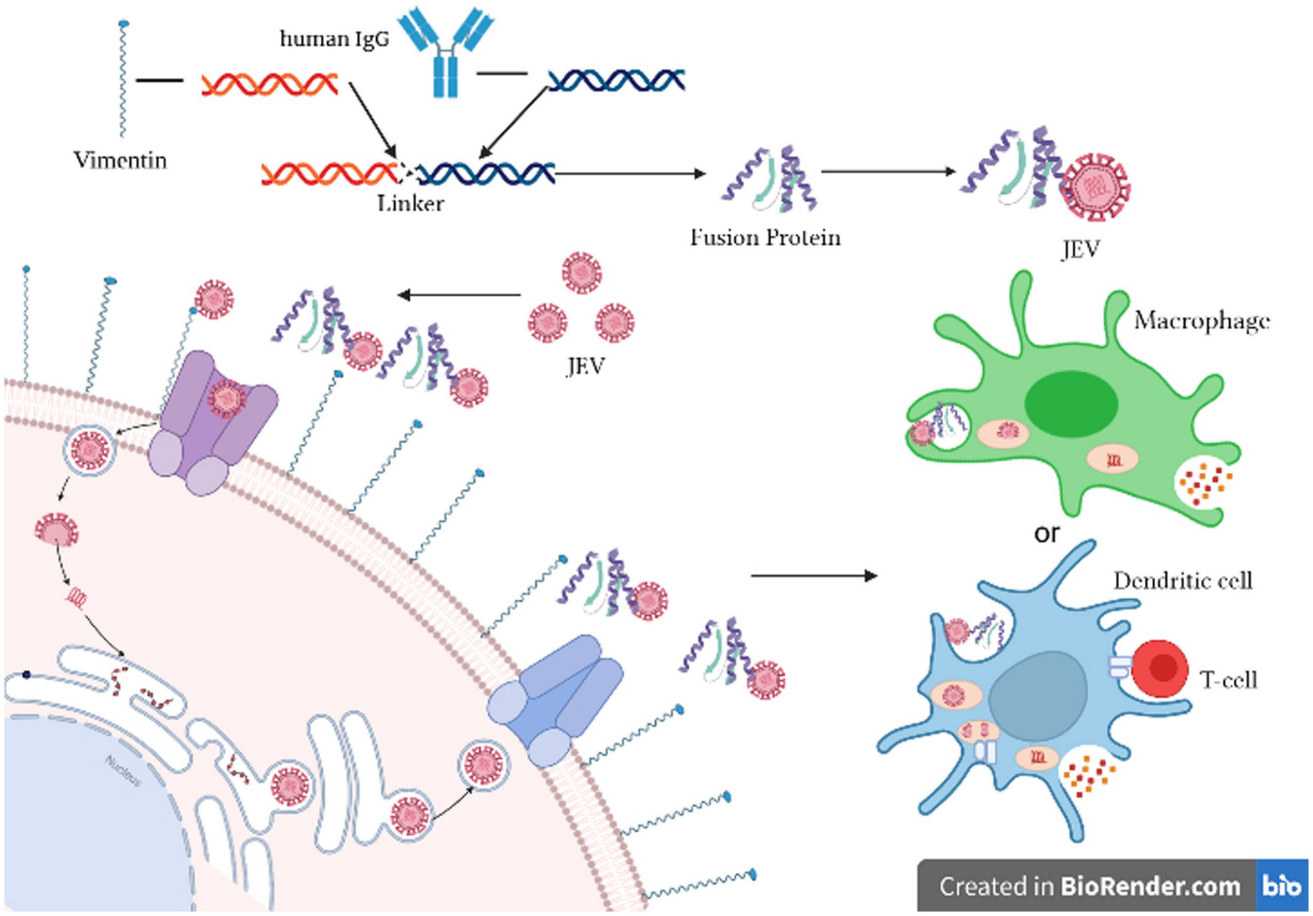
Schematic diagram of the inhibition of JEV by vimentin-Fc fusion protein. Created in Biorender.com.

As illustrated in Figure 1, the experimental principle involves cloning the cell wave protein of vimentin and the Fc fragment of human IgG. These two genes are linked using a linker and expressed as vimentin-Fc fusion protein in *E. coli*. Upon infection of cells with JEV, the vimentin-Fc fusion protein binds to the virus, effectively preventing its attachment to receptor sites on target cell membranes and impeding random virus internalization. In the case of JEV infection in an organism, the vimentin-Fc fusion protein not only binds to the virus but also localizes through the Fc fragment. This localization enables recognition by immune cells such as phagocytes or dendritic cells, leading to their killing or phagocytosis.

### 3.2 Specificity validation of monoclonal antibody against JEV

**Figure 2:**
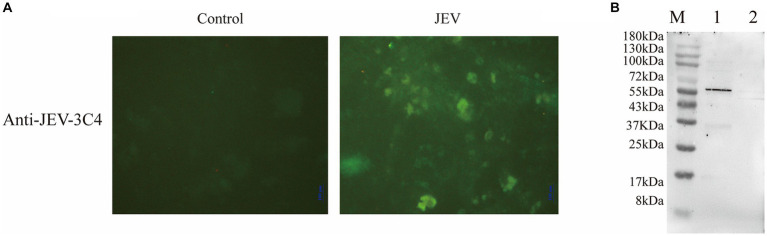
Validation of monoclonal antibody against JEV. **(A)** Specificity validation of monoclonal antibody 3C4 against JEV using indirect immunofluorescence. Indirect immunofluorescence was employed to determine the specificity of the antibody towards JEV-infected Vero cells compared to uninfected normal Vero cells (control). **(B)** Specificity validation of monoclonal antibody 3C4 against JEV using western blotting. BHK-21 cells infected with JEV were lysed using RIPA buffer, and the resulting cell lysate was collected for protein analysis. The 3C4 antibody was used as the primary antibody in the protein blotting procedure. Uninfected normal BHK-21 cells were utilized as a negative control. M: 180 kDa; Lane 1: JEV-infected BHK-21 cells; Lane 2: Normal BHK-21 cells.

In this study, we employed a suckling mice brain tissue suspension that had been challenged by the virus as the immunogen to immunize mice and generate multiple monoclonal antibodies targeting the virus. Successful immunization led to the fusion of mouse spleen cells with myeloma cells, followed by three rounds of screening to identify highly potent and stable positive monoclonal antibodies. By employing an indirect immunofluorescence assay, we compared JEV-infected and non-infected cells to assess the specificity of the JEV-3C4 monoclonal antibody, revealing a significant contrast in fluorescence between the two groups (Figure 2A).

To ascertain the specificity of JEV-3C4 monoclonal antibody towards JEV viral proteins, BHK-21 cells infected with JEV for 72 h were lysed using RIPA buffer, and total cellular proteins were extracted. Uninfected BHK cells were used as a negative control. The processed cell protein samples were subjected to SDS-PAGE, followed by transfer onto a membrane. For western blot validation, JEV-3C4 monoclonal antibody was employed as the primary antibody, while goat anti-mouse IgG-HRP served as the secondary antibody. Figure 2B illustrates the specific reactivity of JEV-3C4 monoclonal antibody with JEV-infected cells, exhibiting no reactivity with uninfected normal BHK cells. Furthermore, the specific band was observed to be below 55 kDa. The isotype of 3C4 is IgM, and the light chain is κ. Therefore, this monoclonal antibody can be used as a marker monoclonal antibody for NS1 protein in JEV and provide material for subsequent WB detection experiments.

### 3.3 Construction of the prokaryotic expression vector

**Figure 3:**
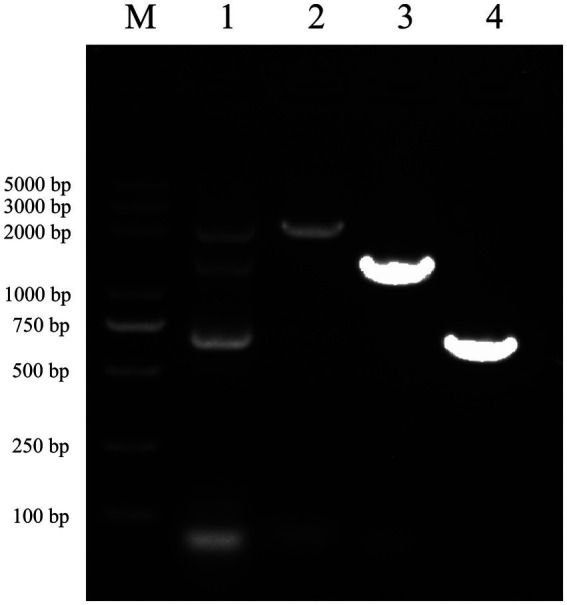
Identification of vimentin-Fc gene by PCR M: DL5000 DNA Marker. (1) The fragments of vimentin-Fc fusion gene, the sizes of the bands were 2,150 bp, 1,400 bp and 750 bp, which were consistent with the expected sizes. (2) The identification of vimentin-Fc gene by PCR. The sizes of the bands were 2,150 bp, which were consistent with the expected sizes. (3) The vimentin gene was amplified by PCR as the vimentin-Fc fusion protein amplification control with a band of 1,400 bp, which is in accordance with the expected results. (4) PCR amplification of the FC fragment gene was used as a control for the vimentin-Fc fusion protein, and the band size was 750 bp, which was consistent with the expected results.

After performing RNA extraction and cDNA synthesis, successful amplification of vimentin and Fc genes was achieved (Figure 3, Lanes 3 and 4). These genes were subsequently ligated using seamless cloning to generate the vimentin-Fc fusion gene (Figure 3, Lane 1). The recombinant plasmid pET-22b-vimentin-Fc was constructed by digesting the pET-22b vector with *EcoR* I and *Not* I restriction enzymes, followed by ligation. Further confirmation of the recombinant plasmid was obtained through DNA sequencing and broth PCR (Figure 3, Lane 2).

### 3.4 Purification of the vimentin-Fc protein

**Figure 4:**
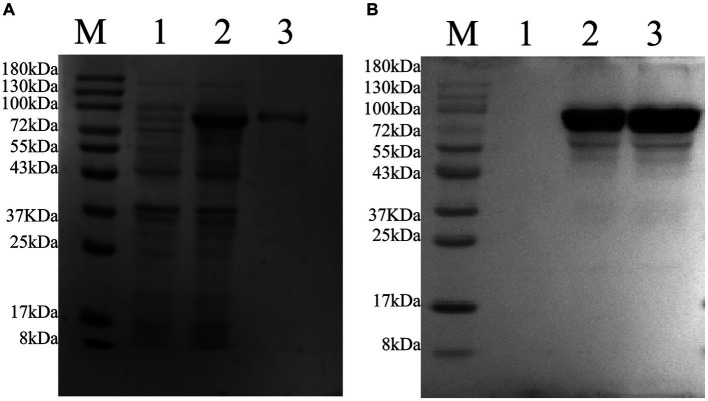
Purification of pET-22b-vimentin-Fc protein, protein size is 75 kDa. M: 180 protein Marker. **(A)** SDS-PAGE analysis of recombinant protein expressed in *E. coli* Rosetta (DE3). (1) *E. coli* Rosetta (DE3) bacterial solution containing pET-22b-vimentin-Fc uninduced. (2) *E. coli* Rosetta (DE3) containing pET-22b-vimentin-Fc was induced with IPTG. (3) pET-22b-vimentin-Fc purified protein. **(B)** Western blot identification of recombinant protein vimentin-Fc. (1) *E. coli* Rosetta (DE3) bacterial solution containing pET-22b-vimentin-Fc uninduced. (2) *E. coli* Rosetta (DE3) containing pET-22b-vimentin-Fc was induced with IPTG. (3) pET-22b-vimentin-Fc purified protein.

After transfecting the recombinant plasmid pET-22b-vimentin-Fc into *E. coli* Rosetta (DE3) cells, the expression of the vimentin-Fc fusion protein was induced by the addition of IPTG. The induction with IPTG resulted in a relatively high expression level of the vimentin-Fc protein (Figure 4A, Lane 2), whereas no expression was observed in the absence of IPTG (Figure 4A, Lane 1). The recombinant vimentin-Fc protein displayed an estimated size of 97.4 kDa, and its band position matched the expected size. Subsequently, all bacteria were collected and subjected to PBS washing before being sonicated on ice. After centrifugation at 12,000 × g and 4°C for 20 min, a pellet was obtained. The pellet was then denatured using 8 M urea and subjected to purification using Ni-NTA agarose resin. The purity of the purified vimentin-Fc protein was confirmed through SDS-PAGE analysis (Figure 4A, Lane 3). Western blot results are depicted in Figure 4B.

### 3.5 Pull-down experiments

The Ni-NTA agarose beads were employed to capture the vimentin-Fc recombinant protein, which features a His tag. Subsequently, the complex underwent washing and incubation with cell culture supernatant obtained from JEV-infected cells, as well as normal cell culture supernatant. In a similar manner, the protein A/G agarose was coupled to the vimentin-Fc recombinant protein via the Fc fragment, followed by subsequent incubations mirroring the Ni-NTA agarose bead protocol.

**Figure 5:**
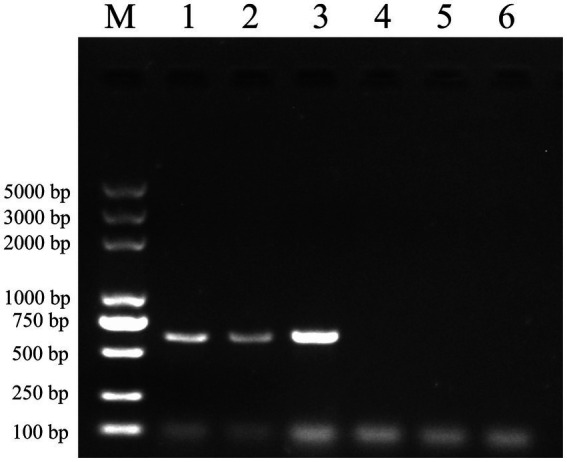
Pull-down experiments: Ni-NTA and protein A/G agarose beads were, respectively, conjugated to the fusion protein and then mixed and incubated with JEV virus culture, and the mixture was extracted for RNA and subjected to PCR amplification for detection of the JEV-M gene. The JEV-M gene is 600 bp in size and the following results are consistent with the expected results. Lane: 1 PCR of protein A/G experimental group after pull-down. Lane 2: PCR of the Ni-NTA experimental group after pull-down. Lane 3: positive control (PCR for JEV virus). Lane 4: PCR of the Ni-NTA control group after pull-down. Lane 5: PCR of the protein A/G control group after pull-down. Lane 6: PCR negative control.

Following the completion of incubation, multiple washes were carried out. RNA was extracted using the Trizol method and reverse transcribed into cDNA, which was then subjected to identification via amplification using specific primers for JEV (M-E). Figure 5 demonstrates the *in vitro* binding capability of the vimentin-Fc protein to JEV. The Ni-NTA-vimentin-Fc complex exhibited binding to JEV (Figure 5, Lane 1), and similarly, the protein A/G-vimentin-Fc captured the JEV virus *in vitro* (Figure 5, Lane 2).

### 3.6 Cytotoxicity assay

**Figure 6:**
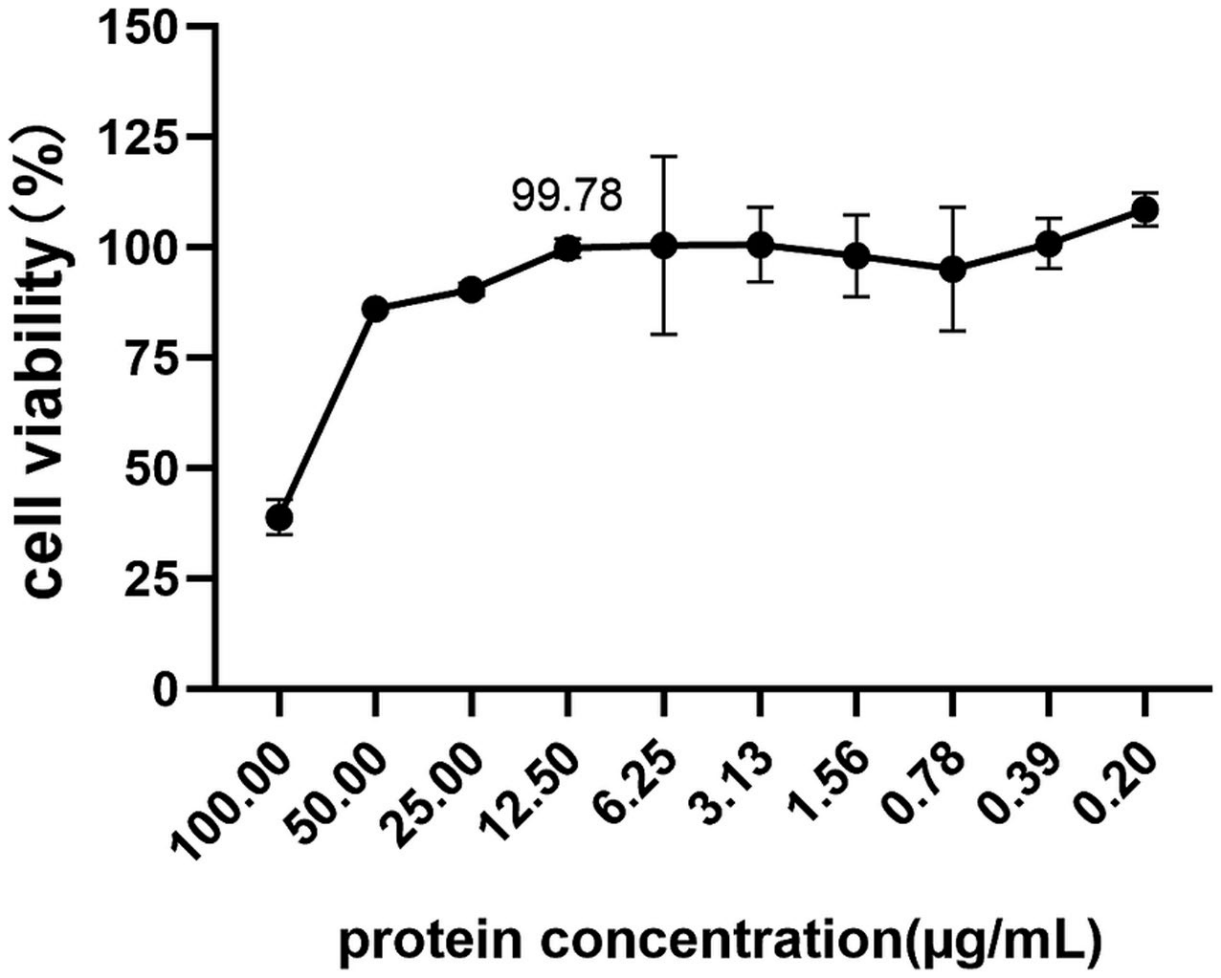
The cytotoxicity of vimentin-Fc protein. BHK-21 cells were treated with vimentin-Fc protein in the concentration range of 0.2–100 μg/mL and incubated for a period of 48 h. The viability of the cells was evaluated utilizing the CCK8 assay, and the cell survival rate was determined by applying a specific formula.

To investigate the cytotoxicity of vimentin-Fc protein on BHK-21 cells, which are susceptible to JEV infection, the cells were treated with vimentin-Fc protein at concentrations ranging from 0.2 to 100 μg/mL and incubated for 48 h. Cell toxicity was analyzed using the CCK8 assay, and the cell survival rate was calculated using a specific formula. Figure 6 demonstrates that when the vimentin-Fc protein concentration was maintained at 12.5 μg/mL, the cell survival rate was 99.78%. At this particular protein concentration, vimentin-Fc protein had negligible impact on BHK-21 cells. Therefore, vimentin-Fc protein at or below a concentration of 12.5 μg/mL can be utilized in subsequent experiments to inhibit JEV viral proliferation.

### 3.7 Inhibition of JEV replication in cells by vimentin-Fc protein

**Figure 7:**
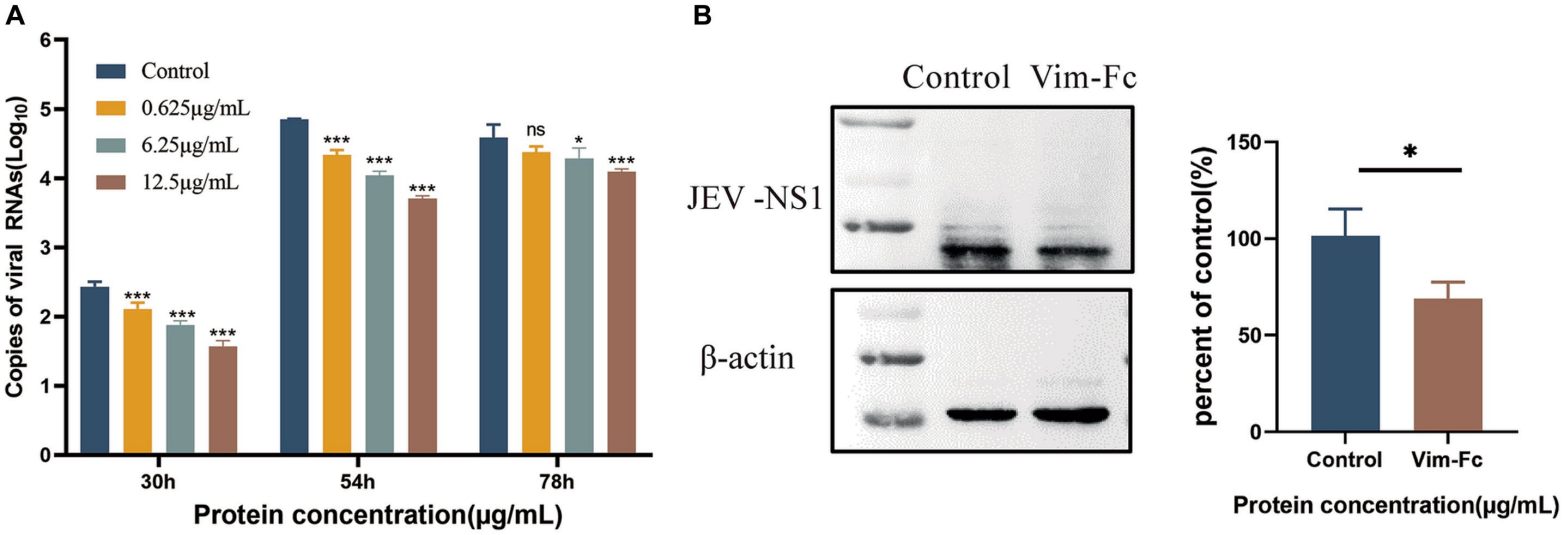
Inhibition of JEV replication in cells by vimentin-Fc protein. **(A)** The RNA copies of JEV virus in the cell culture supernatant at various time points were quantified using qPCR. BHK-21 cells were infected with JEV (MOI = 0.01) and treated with vimentin-Fc fusion protein at concentrations of 0.625 μg/mL, 6.25 μg/mL, and 12.5 μg/mL. The supernatant was collected at 30, 54, and 72 h, and the RNA was extracted for subsequent qPCR analysis to determine the JEV viral copy numbers. **(B)** BHK-21 cells were infected with JEV (MOI = 0.01) and treated with 12.5 μg/mL of vimentin-Fc protein for 72 h. The cell lysate was collected, and protein immunoblotting was performed using antibodies against JEV-NS1 or β-actin. The protein band intensities were quantified using ImageJ software, and the relative ratio of JEV-NS1 to actin was calculated. The data are presented as the mean ± standard error of the mean (SEM). Statistical significance was determined using student’s *t*-test (^*^p < 0.05, ^**^p < 0.01, ^***^p < 0.001, and ^****^p < 0.0001). Each group was analyzed at least three times.

To investigate the impact of vimentin-Fc protein on the binding of JEV to host cells following infection, vimentin-Fc protein or BSA was added to BHK cells. Culture supernatant and cell lysates from infected cells were collected, and virus loads were determined using real-time fluorescence quantitative PCR and protein blotting. Figure 7A reveals that vimentin-Fc protein exerts a significant inhibitory effect on JEV infection of host cells and subsequent virus replication. Maintaining concentrations of 0.625 μg/mL, 6.25 μg/mL, and 12.5 μg/mL of vimentin-Fc protein during 30 h and 54 h of JEV infection resulted in a remarkable reduction in JEV proliferation. However, with the passage of time, at 72 h, the inhibitory effect of vimentin-Fc protein at concentrations of 0.625 μg/mL and 6.25 μg/mL was not as pronounced as that observed with 12.5 μg/mL. Further confirmation of the inhibitory effect of vimentin-Fc protein on JEV replication was obtained at the viral protein level. Cell lysates from cells treated continuously with 12.5 μg/mL of vimentin-Fc protein after JEV infection were collected and subjected to Western blot analysis alongside the control group. As depicted in Figure 7B, the abundance of viral NS3 protein in vimentin-Fc protein-treated cells (JEV + vimentin-Fc) was significantly lower compared to untreated cells (JEV + PBS) after 72 h of infection.

## 4 Discussion

JEV and other viruses from the Flaviviridae family have the ability to penetrate the blood-brain barrier and infect the central nervous system, resulting in viral encephalitis. Vimentin proteins and other cytoskeletal filaments play significant roles in many viral replication and infection processes. It has been reported that vimentin protein is involved in the binding and infection of highly virulent strains of JEV, and monoclonal antibodies against wave proteins can inhibit JEV entry. In this study, we capitalized on the interaction between vimentin protein and JEV viral proteins to create a recombinant dimeric protein (vimentin-Fc) by fusing the cDNA of vimentin protein with the Fc region of human IgG1. The vimentin segment of vimentin-Fc corresponds to the Fab region of IgG. The successful fusion of the vimentin-Fc gene with the pET-22b vector led to the construction of the recombinant pET-22b-vimentin-Fc, which was subsequently expressed in *E. coli*. Purification of the expressed protein yielded the vimentin-Fc fusion protein. Pull-down experiments demonstrated that Ni-NTA agarose beads efficiently captured the His-tagged pET-22b-vimentin-Fc, while coupling with protein A/G and the Fc fragment allowed binding with JEV *in vitro*. In cell-based assays investigating the inhibitory effects of vimentin-Fc on JEV infection, the presence of 12.5 μg/mL vimentin-Fc protein in the cell culture medium significantly suppressed JEV proliferation at 30 h, 54 h and 72 h, indicating sustained effectiveness. Similarly in the experiment, the low dose of fusion protein (0.625 μg/mL) lost its significance in the later stages of viral infection, probably because as more virus was added, the binding of the fusion protein became saturated and could not stand in the way of inhibiting further viral proliferation.

Vimentin protein is also expressed on the cell surface and can even be secreted into the extracellular environment by activated macrophages. It has been demonstrated to play a role in viral transport to the cell membrane and subsequent release (25, 26). Fc fusion proteins have gained approval as the sole class of fusion protein products in biopharmaceuticals due to their ability to facilitate cell uptake through surface Fc receptors. Examples such as Etanercept (Enbrel) for rheumatoid arthritis and Abatacept (Orencia), a tumor necrosis factor inhibitor, have achieved clinical success (27). Fc fusion proteins are one of the most successful methods of prolonging protein half-life in the clinical setting, and Fc protein mediated prolongation of IgG1 half-life improved the antitumour activity of Fc-engineered antibodies in mice (28), suggesting that, at least in these mouse models of cancer, increasing drug exposure by prolonging half-life improves efficacy and reduces the frequency of drug administration (29). In this experiment, the constructed vimentin-Fc fusion protein, by binding to JEV via vimentin, exhibits potential as a novel antiviral drug against JEV. However, the expression of the vimentin-Fc construct in prokaryotic bacteria lacks the control of expression time and level. Prolonged expression of certain genes can be detrimental to host cells, and maintaining the protein in a prokaryotic environment may cause damage to eukaryotic cells. Therefore, an appropriate dosage needs to be determined to maintain effective inhibition of JEV replication while ensuring maximal cell proliferation and minimizing potential cytotoxicity.

Future research will involve *in vivo* experiments to investigate the potential significance of vimentin-Fc uptake in inhibiting JEV replication. The preliminary validation of this experiment showed that the fusion protein was able to inhibit JEV in a certain extent, so future research is expected to replace it with the eukaryotic expression system. Eukaryotic cells such as Chinese Hamster Ovary (CHO) cells were chosen to ensure correct translation and folding of the fusion protein and to increase the conformational naturalness of the fusion protein expression. Fusion proteins expressed in eukaryotic systems may show better drug effects *in vivo*. Previous studies have suggested that vimentin acts as a significant receptor or potential receptor, facilitating viral entry into the blood-brain barrier and exacerbating infection. Further investigations in this area can shed light on the antiviral mechanisms and the feasibility of developing antiviral drugs or therapeutic vaccines for clinical applications.

## Data availability statement

The original contributions presented in the study are included in the article/supplementary material, further inquiries can be directed to the corresponding authors.

## Ethics statement

The animal study was approved by Experimental Animal Ethics Committee of Kunming University of Science and Technology. The study was conducted in accordance with the local legislation and institutional requirements.

## Author contributions

TZ: Conceptualization, Writing – review & editing, Methodology, Validation, Writing – original draft. ZC: Methodology, Writing – original draft, Writing – review & editing, Formal analysis. LX: Writing – review & editing, Methodology, Validation, Writing – original draft. RX: Writing – review & editing, Writing – original draft, Methodology, Validation. LC: Methodology, Writing – review & editing. TJ: Writing – review & editing. WS: Writing – review & editing. YW: Writing – review & editing. YS: Writing – review & editing. QH: Writing – review & editing. XX: Writing – review & editing. TY: Writing – review & editing, Funding acquisition. JZ: Conceptualization, Funding acquisition, Supervision, Writing – original draft, Writing – review & editing.

## References

[1] Sumiyoshi H Hoke CH Trent DW. Infectious Japanese encephalitis virus RNA can be synthesized from *in vitro*-ligated cDNA templates. J Virol. (1992) 66:5425–31. doi: 10.1128/jvi.66.9.5425-5431.1992, PMID: 1501281 PMC289099

[2] Rosen L. The natural history of Japanese encephalitis virus. Ann Rev Microbiol. (1986) 40:395–414. doi: 10.1146/annurev.mi.40.100186.002143, PMID: 2877613

[3] Weaver SC Barrett ADT. Transmission cycles, host range, evolution and emergence of arboviral disease. Nat Rev Microbiol. (2004) 2:789–801. doi: 10.1038/nrmicro1006, PMID: 15378043 PMC7097645

[4] Kumar R Tripathi P Singh S Bannerji G. Clinical features in children hospitalized during the 2005 epidemic of Japanese encephalitis in Uttar Pradesh, India. Clin Infect Dis. (2006) 43:123–31. doi: 10.1086/505121, PMID: 16779737

[5] Martina BE Koraka P van den Doel P van Amerongen G Rimmelzwaan GF Osterhaus ADME. Immunization with West Nile virus envelope domain III protects mice against lethal infection with homologous and heterologous virus. Vaccine. (2008) 26:153–7. doi: 10.1016/j.vaccine.2007.10.055, PMID: 18069096 PMC7127062

[6] Murgod UA Muthane UB Ravi V Radhesh S Desai A. Persistent movement disorders following Japanese encephalitis. Neurology. (2001) 57:2313–5. doi: 10.1212/wnl.57.12.2313, PMID: 11756619

[7] Pierson TC Diamond MS. Degrees of maturity: the complex structure and biology of flaviviruses. Curr Opin Virol. (2012) 2:168–75. doi: 10.1016/j.coviro.2012.02.011, PMID: 22445964 PMC3715965

[8] Chokephaibulkit K Houillon G Feroldi E Bouckenooghe A. Safety and immunogenicity of a live attenuated Japanese encephalitis chimeric virus vaccine (IMOJEV^®^) in children. Expert Rev Vaccines. (2015) 15:153–66. doi: 10.1586/14760584.2016.1123097, PMID: 26588242

[9] Barzon L Palù G. Recent developments in vaccines and biological therapies against Japanese encephalitis virus. Expert Opin Biol Ther. (2018) 18:851–64. doi: 10.1080/14712598.2018.1499721, PMID: 29991325

[10] Beasley DW Lewthwaite P Solomon T. Current use and development of vaccines for Japanese encephalitis. Expert Opin Biol Ther. (2007) 8:95–106. doi: 10.1517/14712598.8.1.95 18081539

[11] Charrier EE Janmey PA. Mechanical properties of intermediate filament proteins. Methods Enzymol. (2016) 568:35–57. doi: 10.1016/bs.mie.2015.09.009, PMID: 26795466 PMC4892123

[12] Liang JJ Yu CY Liao CL Lin YL. Vimentin binding is critical for infection by the virulent strain of Japanese encephalitis virus. Cell Microbiol. (2011) 13:1358–70. doi: 10.1111/j.1462-5822.2011.01624.x, PMID: 21707907

[13] Cogli L Progida C Bramato R Bucci C. Vimentin phosphorylation and assembly are regulated by the small GTPase Rab7a. Biochim Biophys Acta. (2013) 1833:1283–93. doi: 10.1016/j.bbamcr.2013.02.024, PMID: 23458836 PMC3787733

[14] Patteson AE Vahabikashi A Goldman RD Janmey PA. Mechanical and non-mechanical functions of filamentous and non-filamentous vimentin. BioEssays. (2020) 42:e2000078–8. doi: 10.1002/bies.202000078, PMID: 32893352 PMC8349470

[15] Sivagurunathan S Vahabikashi A Yang H Zhang J. Expression of vimentin alters cell mechanics, cell–cell adhesion, and gene expression profiles suggesting the induction of a hybrid EMT in human mammary epithelial cells. Front Cell Dev Biol. (2022) 10:929495. doi: 10.3389/fcell.2022.929495, PMID: 36200046 PMC9527304

[16] Zhou Z Cui F Wen Q Zhou H. Effect of vimentin on cell migration in collagen-coated microchannels: a mimetic physiological confined environment. Biomicrofluidics. (2021) 15:034105. doi: 10.1063/5.0045197, PMID: 34025897 PMC8133791

[17] Su L Pan P Yan P Long Y. Role of vimentin in modulating immune cell apoptosis and inflammatory responses in sepsis. Sci Rep. (2019) 9:5747. doi: 10.1038/s41598-019-42287-7, PMID: 30952998 PMC6451033

[18] Meng Y Lin S Niu K Ma Z Lin H Fan H. Vimentin affects inflammation and neutrophil recruitment in airway epithelium during *Streptococcus suis* serotype 2 infection. Vet Res. (2023) 54:7. doi: 10.1186/s13567-023-01135-3, PMID: 36717839 PMC9885403

[19] Du N Cong H Tian H Zhang H. Cell surface vimentin is an attachment receptor for enterovirus 71. J Virol. (2014) 88:5816–33. doi: 10.1128/JVI.03826-13, PMID: 24623428 PMC4019121

[20] Kim D Lee SH Na K. Immune stimulating antibody-photosensitizer conjugates via Fc-mediated dendritic cell phagocytosis and phototriggered immunogenic cell death for KRAS-mutated pancreatic cancer treatment. Small. (2021) 17:e2006650–09. doi: 10.1002/smll.202006650, PMID: 33590726

[21] Hao Y Tang X Xing J Sheng X Chi H Zhan W. Regulatory role of Fc receptor in mIgM^+^ B lymphocyte phagocytosis in flounder (*Paralichthys olivaceus*). Front Immunol. (2021) 12:804244. doi: 10.3389/fimmu.2021.804244, PMID: 34975918 PMC8718553

[22] Andersen JT Foss S Kenanova VE Olafsen T. Anti-carcinoembryonic antigen single-chain variable fragment antibody variants bind mouse and human neonatal Fc receptor with different affinities that reveal distinct cross-species differences in serum half-life. J Biol Chem. (2012) 287:22927–37. doi: 10.1074/jbc.m112.355131, PMID: 22570488 PMC3391105

[23] Zhang J Ruan X Zan J Zheng X. Efficient generation of monoclonal antibodies against major structural proteins of rabies virus with suckling mouse brain antigen. Monoclon Antib Immunodiagn Immunother. (2014) 33:94–100. doi: 10.1089/mab.2013.0087, PMID: 24746150

[24] Xu X Chen G Huang Y Ding L. Development of multiplex PCR for simultaneous detection of six swine DNA and RNA viruses. J Virol Methods. (2012) 183:69–74. doi: 10.1016/j.jviromet.2012.03.034, PMID: 22575688

[25] Zhang Y Zhao S Li Y Feng F. Host cytoskeletal vimentin serves as a structural organizer and an RNA-binding protein regulator to facilitate Zika viral replication. Proc Natl Acad Sci U S A. (2022) 119:e2113909119. doi: 10.1073/pnas.2113909119 35193960 PMC8872754

[26] Ramos I Stamatakis K Oeste CL Pérez-Sala D. Vimentin as a multifaceted player and potential therapeutic target in viral infections. Int J Mol Sci. (2020) 21:4675. doi: 10.3390/ijms21134675, PMID: 32630064 PMC7370124

[27] Jafari R Zolbanin NM Rafatpanah H Majidi J Kazemi T. Fc-fusion proteins in therapy: an updated view. Curr Med Chem. (2017) 24:1228–37. doi: 10.2174/0929867324666170113112759, PMID: 28088904

[28] Sockolosky JT Szoka FC. The neonatal Fc receptor, FcRn, as a target for drug delivery and therapy. Adv Drug Deliv Rev. (2015) 91:109–24. doi: 10.1016/j.addr.2015.02.005, PMID: 25703189 PMC4544678

[29] Zalevsky J Chamberlain AK Horton HM Karki S Leung IWL Sproule TJ. Enhanced antibody half-life improves *in vivo* activity. Nat Biotechnol. (2010) 28:157–9. doi: 10.1038/nbt.1601, PMID: 20081867 PMC2855492

